# An Innovative Simple Electrochemical Levofloxacin Sensor Assembled from Carbon Paste Enhanced with Nano-Sized Fumed Silica

**DOI:** 10.3390/bios12100906

**Published:** 2022-10-21

**Authors:** Amany M. Fekry

**Affiliations:** Chemistry Department, Faculty of Science, Cairo University, Giza 12613, Egypt; famany@cu.edu.eg; Tel.: +20-2-0101545331

**Keywords:** levofloxacin, nano-sized fumed silica, EIS, SEM, EDX

## Abstract

A new electrochemical sensor for the detection of levofloxacin (LV) was efficiently realized. The aim was to develop a new, cheap, and simple sensor for the detection of LV, which is used in various infections due to its pharmacological importance. It consists of carbon paste (CP) enhanced with nano-sized fumed silica (NFS). NFS has a very low bulk density and a large surface area. The carbon paste-enhanced NFS electrode (NFS/CPE) showed great electrocatalytic activity in the oxidation of 1.0 mM LV in Britton–Robinson buffer (BR) at pH values ranging from 3.0 to 8.0. Cyclic voltammetry (CV) and differential pulse voltammetry (DPV) were used; the peak current value (I_p_) of the NFS/CPE sensor was 2.7 times that of the bare electrode, ensuring its high electrocatalytic activity. Electrochemical impedance spectroscopy (EIS) was performed at a peak potential (E_p_) of +1066 mV, yielding a resistance of 10 kΩ for the designed NFS/CPE sensor compared to 2461 kΩ for the bare electrode, indicating the high conductivity of the modified sensor and verifying the data observed using the CV technique. Surface descriptions were determined by scanning electron microscopy (SEM) and energy dispersive X-ray analysis (EDX). The variation in the concentration of LV (2.0 to 1000 µM) was considered in BR buffer (pH = 5.0) at a scan rate (SR) of 10 mV/s by the NFS/CPE. The detection and quantification limits were 0.09 µM and 0.30 µM, respectively. To evaluate the application of LV in real samples, this procedure was established on Quinostarmax 500 mg tablets and human plasma samples. Reasonable results were obtained for the detection of LV.

## 1. Introduction

Antibiotics are widely used to treat various diseases to promote human and animal health [1]. Levofloxacin (LV) is among the fourth generation of fluoroquinolone (FQs) antibiotics which are used for bacterial infections in various sites of the human body, especially in the respiratory tract, genitourinary structure, and soft tissues of the skin. LV has fewer toxic side effects than other antibacterial drugs [2,3,4].

Antibiotics such as LV are extensively used in agriculture and aquaculture [5]. Therefore, a sensitive and discriminatory system for the discovery of LV is essential for both human health and the environment. The methods used to screen these analytes are spectrophotometry [6,7], high-performance liquid chromatography (HPLC) [8,9], chemiluminescence [10,11], and electrochemical devices [12,13].

Electrochemical approaches are simpler and more sensible, with outstanding repeatability, short examination times, and little in terms of expense in comparison to other conventional approaches. Additionally, modification promotes the existence of electrochemical reactions by fluctuating the oxidation and/or reduction potential values [14]. This avoids some of the problems of surface contamination [15,16].

Studies of nanoparticles are of great importance due to their chemical and physical properties [17]. Electroactive nanoparticles are extensively used for the construction of electrochemical sensors for the discovery of various substances. Nano-sized fumed silica (NFS) is prepared by a flame process and contain silica groups in the form of agglomerates of forked and chain-like three-dimensional particles. The FS comprises several silanol groups that react with various constituents. Its surface is non-porous due to the arrangement of an amorphous structure, and it has a high surface area due to its nanostructure [17,18] and low density. Therefore, it is widely used in many fields and can be used in catalysis, coatings, sensors, and other applications [19]. FS can be considered an excellent modifier in the fabrication of adapted electrodes and a noteworthy material for electrochemical sensing trials [14,20]. Furthermore, it can enhance the molecular interactions on the electrode surface through silanol groups (Si-OH). Therefore, NFS can be used to fabricate a modified carbon paste electrode.

The usual dose of a levofloxacin injection is 250 mg or 500 mg managed by slow infusion over 60 min every 24 h or 750 mg managed by slow infusion over 90 min every 24 h, depending on the infection type, such as nosocomial pneumonia, complicated skin and skin structure infections, acute bacterial sinusitis, and others [21].

For interfering compounds, paracetamol (APAP) is a worldwide analgesic and antipyretic drug used to treat a variable number of illnesses such as fevers, coughs, colds, and aches. Recently, it was used to reduce fevers and control the inflammation in SARS-COVID-19 patients. Thus, the detection of APAP is essential, as it is needed with other drugs [22,23]. Urea in the human body is the final product of the protein metabolism, which is mainly discharged through the kidney with urine. It can be transported through cell membrane diffusion to the blood, saliva, reproductive organs, and breasts [24]. Thus, it is significant and needs to be detected.

Recently, LV has been of paramount pharmacological importance, which is being used to deal with a number of bacterial infections. Thus, the creation of a new sensor for its detection, especially in tablets and human plasma, is required, one that is an effective sensor to determine LV in both cases. This would make the sensor useful in marketing for applications. To the best of my knowledge, there are few electrochemical works on LV in the literature. Thus, LV examination by an innovative, i.e., a lower detection limit, sensor compared to others and rapid and simple methods compared to older chemical ones is desired. The goal of this study was to create a simple, lightweight, and inexpensive nano-sized fumed silica improved carbon paste electrode (NFS/CPE) as a sensitive and selective LV sensor. The resulting sensor is cost-effective, detecting concentrations reaching 0.09 µM owing to its large surface area because of its nano-size and amorphous structure that increases its sensitivity. Cyclic and differential pulse voltammetry and impedance measurements were used to accomplish this work. SEM and EDX analyses were performed to explore the surface morphology. The selectivity and sensibility were demonstrated in real samples.

## 2. Materials and Methods

### 2.1. Materials

Levofloxacin (C_18_H_20_FN_3_O_4_) (Hikma Pharmaceuticals company) 1 New Burlington Place, London, W1S 2HR, UK, paracetamol (C_8_H_9_NO_2_), Quinostarmax 500 mg tablets (Pharmacy in Egypt), and graphite microparticles (<50 μm) were acquired from Merck. Glucose (C_6_H_12_O_6_), sucrose (C_12_H_22_O_11_), starch (C_6_H_10_O_5_)_n_, and urea (NH_2_CONH_2_) were acquired from MISR-Scientific Company. Paraffin oil and NFS (SiO_2_) powder (<100 nm average particle size) were acquired from Sigma-Aldrich.

Britton–Robinson (BR) buffer solution (0.04 M boric acid, 0.04 M phosphoric acid, and 0.04 M acetic acid) (pH 3.0–8.0) [17] was used to prepare levofloxacin standard solutions, and the pH was adjusted with 0.2 M NaOH. Triple distilled water was used for all preparations. All experiments were carried out at room temperature.

### 2.2. NFS/CPE Preparation

CPE was performed by the addition of 0.5 g graphite powder to paraffin oil drops, mixing them using a mortar, and then adding 40 mg of NFS by mixing well using a mortar to synthesize the sensor (NFS/CPE) paste. After that, this paste was used to fill a Teflon tube and then pressed onto smooth filter paper until it had a smooth surface. This electrode was used as the synthesized new sensor, the NFS/CPE.

### 2.3. Instruments

A three-electrode cell was used, holding the NFS/CPE as a working electrode (WE), a platinum rod as a counter electrode, and the saturated calomel electrode (SCE) as a reference one [17]. Measurements of CV, DPV, and EIS (10 mV excitation ac amplitude at a frequency range of 0.1 Hz to 100 KHz) were accomplished by the SP-150 potentiostat delivered with the EC-Lab^®^ software package [17]. CV and DPV were carried out to identify the peak current and potential of the sensor. EIS is carried out to recognize the impedance value from which the conductivity was estimated for the sensor. A pH-meter (Hanna Instruments, Italy) was utilized for measuring pH values. Scanning electron microscopy (SEM) measurements were accomplished with the Quanta 250 FEG (Field Emission Gun) combined with an EDX (Energy Dispersive X-ray Analyses) unit (FEI Company, Netherlands). Transmission electron microscopy (TEM) was accomplished using the JEM-1400 electron microscope (JEOL, Japan). This was all carried out to estimate the surface properties of NFS.

### 2.4. Real Samples Preparation

Medicinal aliquots of 500 mg LV pills were squashed in 250 mL of distilled water, stirred magnetically for 30 min, and then settled down and decanted. Then, 2.5 mL of this solution was added to 22.5 mL of BR buffer (pH 5.0) in the electrochemical cell, and the analysis was carried out by using the standard addition method [18].

Blood serum samples were acquired from a laboratory for analysis (Makkah Lab) after centrifugation at 5000 rpm for 5 min to isolate any solid particles and gain a pure serum. Then, 1 mL of the pure serum sample was added to 24 mL of BR buffer (pH 5.0) and sealed into the cell to measure the contents of LV through the standard addition method [18].

## 3. Results and Discussion

### 3.1. Characterization of the NFS/CPE

**Figure 1:**
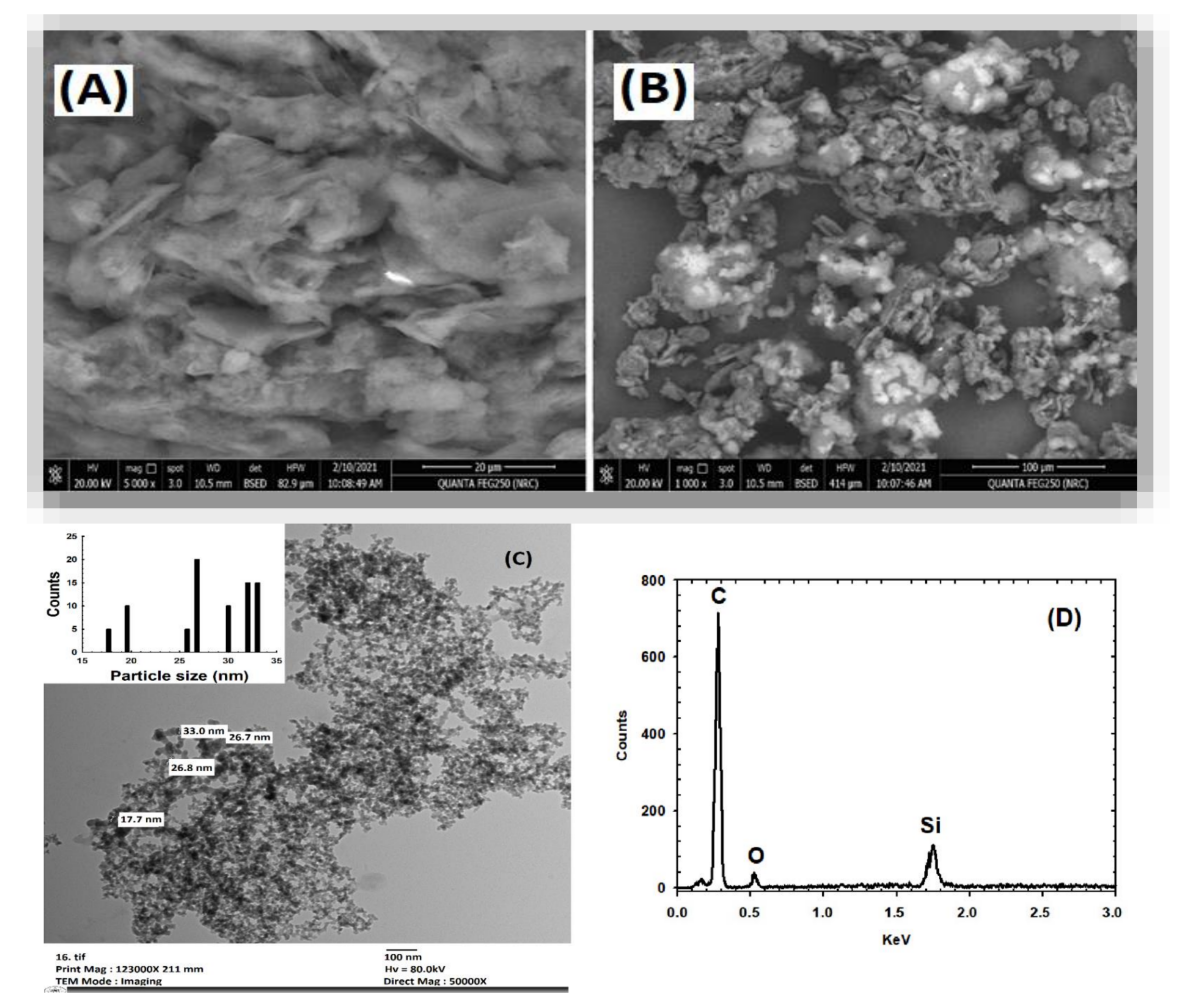
SEM images of (**A**) CPE and (**B**) NFS/CPE electrodes; (**C**) TEM image (size distribution histogram as inset); and (**D**) EDX spectra of nano-sized fumed silica improved carbon paste electrode.

Figure 1A displays the carbon paste electrode (CPE) as a fully exfoliated graphite covered surface. Figure 1B reveals an SEM image of the nano-sized fumed adapted carbon paste electrode (NFS/CPE) at 2000× magnification. The hyperbranched nanofiber structure can be clearly seen in the SEM image [4,18]. In general, the size of the fumed silica nanoparticles is less than 100 nm, as shown in Figure 1C of the TEM image, and the size distribution is clear in the histogram (inset), which shows an electrode with a nanostructure size with a larger surface area than the bare equivalent due to its nanostructure [17,18]. EDX analysis (Figure 1D) confirmed the existence of carbon (C), silicon (Si), and oxygen (O) peaks.

### 3.2. Electrochemistry of Levofloxacin

**Figure 2:**
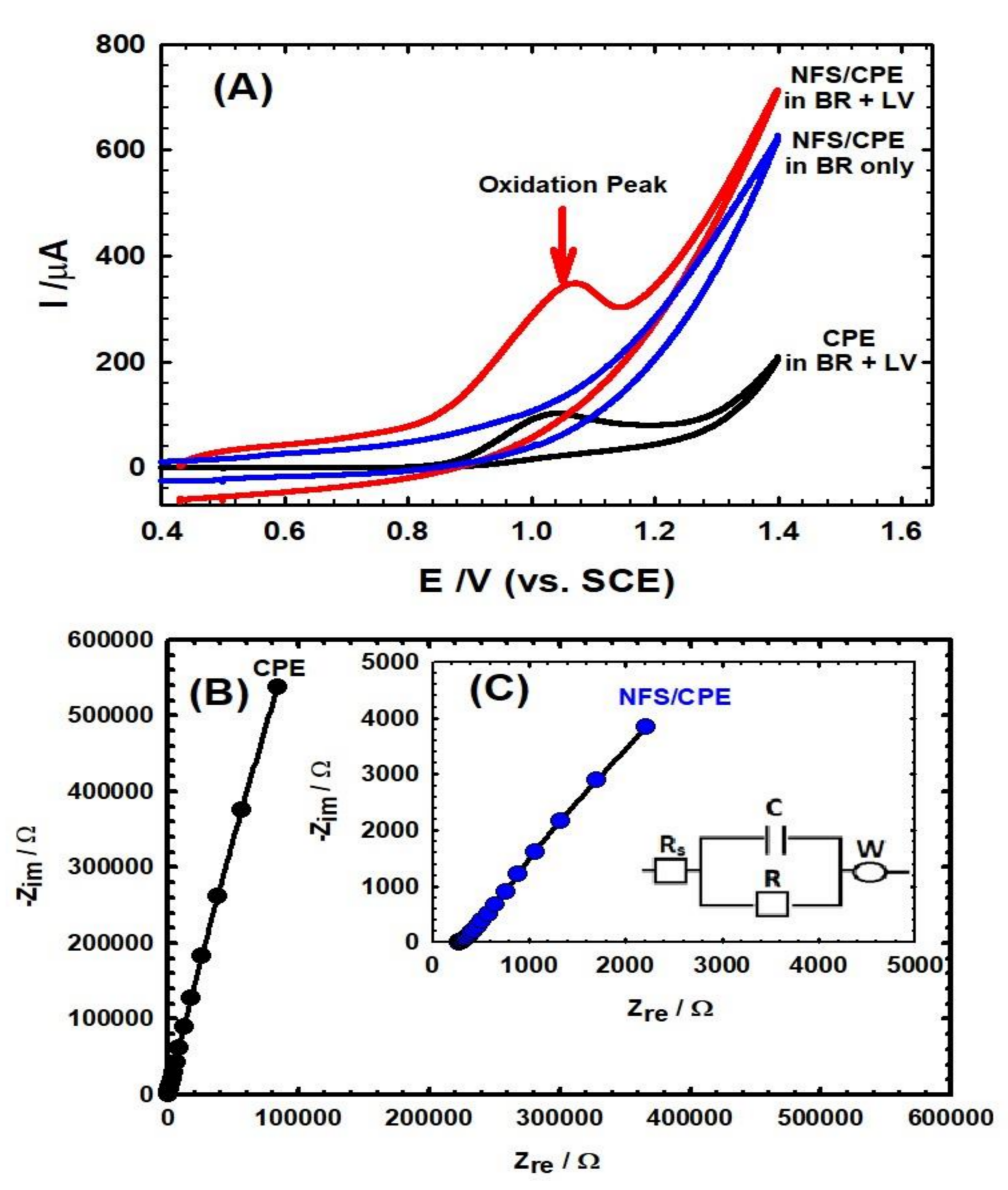
(**A**) CVs (SR = 50 mV/s) and Nyquist plots (at peak potential) of 1 mM LV using BR buffer (pH 5.0) at (**B**) CPE and (**C**) NFS/CPE. **Inset C:** fitting model.

The interaction of 1.0 mM LV with NFS was considered by means of CV. Figure 2A shows the CVs of the CPE and NFS/CPE in BR buffer solution (pH = 5.0) with and/or without LV (no oxidation peak appears) at a SR of 50 mV/s. LV on the NFS/CPE offered a distinct irreversible oxidation peak at +1066 mV, with a peak current (I_p_) ~ 2.7-fold that of CPE in BR buffer containing LV at a 1.0 mM concentration. This shows that NFS can accelerate the electrochemical reaction at the surface of the adapted electrode and contribute significantly to the development of the electrochemical mode of the anticipated sensor. The results show that advanced catalytic activity was attained with NFS.

Electrochemical impedance plots at a peak potential (E_p_) of 1066 mV are shown as Nyquist plots for both bare CPE (Figure 2B) and NFS/CPE (Figure 2C) electrodes. They show a straight line, signifying that the route is principally diffusion-dependent [25]. The model used to fit the experiments by the EC-Lab^®^ software (Figure 2C inset) is a one-time constant exemplary of R_s_ (solution resistance), R (outer layer resistance), W (Warburg impedance due to diffusion), and C (capacitance of outer layer) [26]. The average error of fitting is 1.30%, which is acceptable. The NFS/CPE electrode yields an R_s_ = 277.6 Ω, a C = 543.7 µF, a W = 1.03 kΩ s^−1/2^, and an R = 10 kΩ and the bare electrode an R_s_ = 219.4 Ω, a CPE = 3.76 µF, a W = 23.5 kΩ s^−1/2^, and an R = 2461 kΩ. These results show that the NFS/CPE electrode used is highly conductive [27] due to its lower resistance value in comparison to the bare electrode and permits a remarkable increase in capacitance [4], which assures higher charge capacity and thus higher conductivity. These outcomes sustain the uppermost peak oxidation current achieved from the CV results for the NFS/CPE electrode [28].

### 3.3. Optimal Settings

#### 3.3.1. Influence of pH

**Figure 3:**
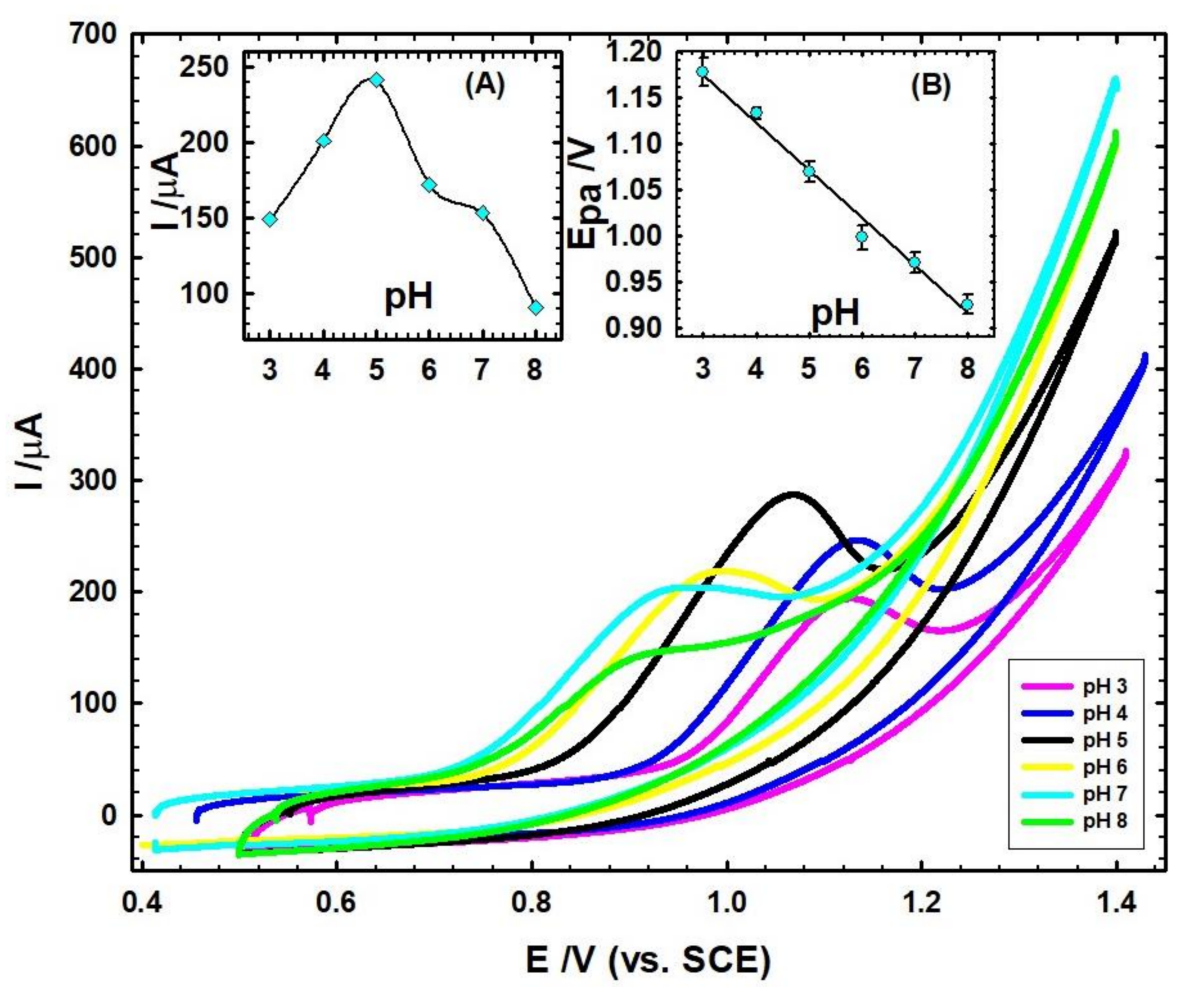
CVs of LV at dissimilar pH values by NFS/CPE, at a scan rate of 50 mV/s. **Inset:** (**A**) Deviation of anodic peak current (I_pa_), and (**B**) potential (E_pa_), with pH at NFS/CPE.

**Scheme 1:**
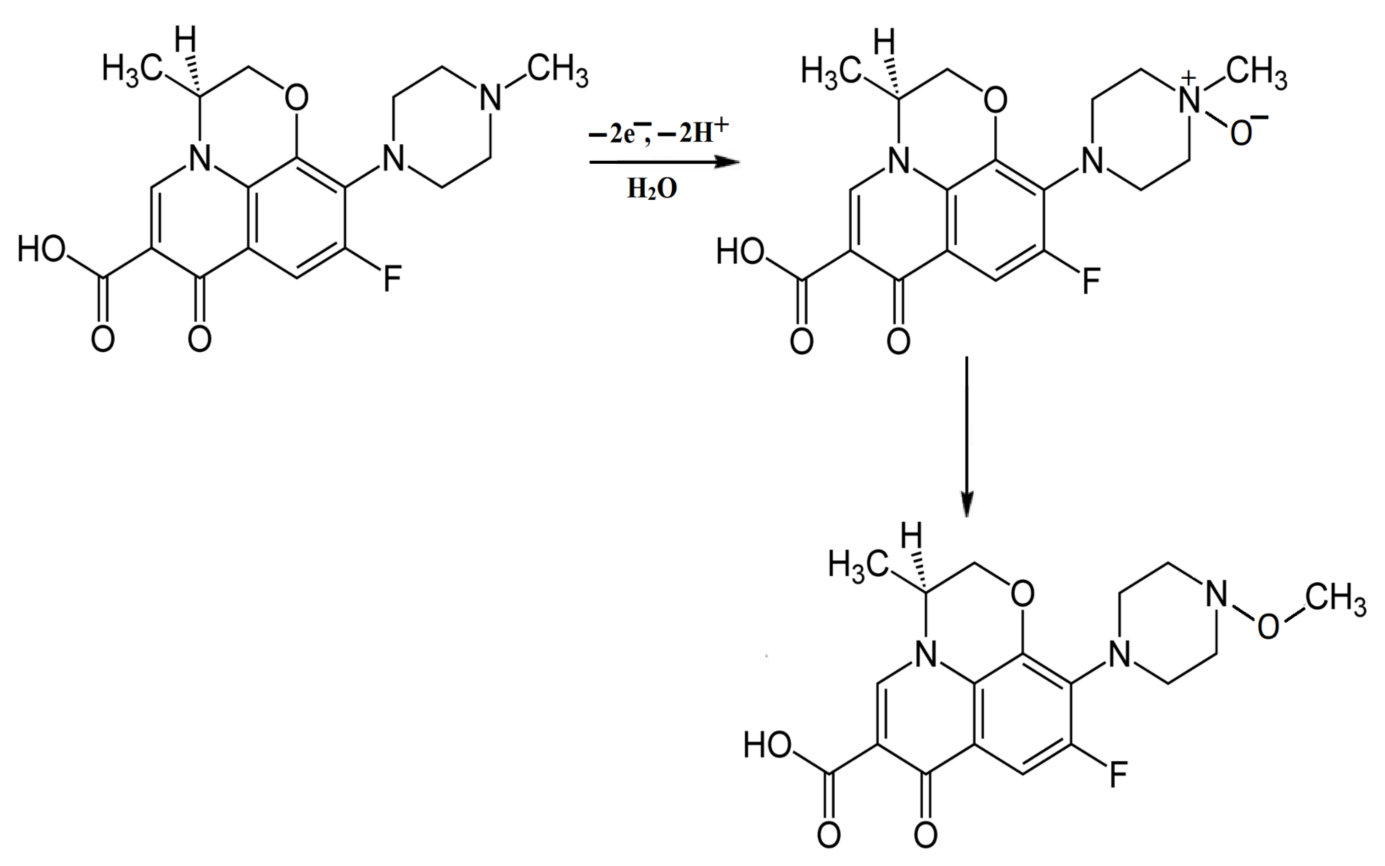
Recommended oxidation reaction mechanism of LV.

The CV was used to determine the outcome of pH alteration on the electrocatalytic oxidation of 1.0 mM LV in BR buffer (pH 3.0–8.0) on the CPE or NFS/CPE (Figure 3). The influence of pH on both the I_p_ and E_p_ for the oxidation of LV can clearly be seen in Figure 3. The E_pa_ shifts negatively and becomes broader with a growing pH. This confirms that the electrocatalytic oxidation of LV is a pH-dependent reaction including an equivalent quantity of protons and electrons. This is consistent with the Nernst equation, according to which E_p_ = 1.33–0.052 pH (R^2^ = 0.98) with a slope of 0.052 (Figure 3 inset), which is close to 0.059. This is consistent with previous studies on the electrochemical reactions of LV [4,29]. The recommended mechanism is given in Scheme 1 [4] for an identical quantity of protons and electrons.

The peak current (I_p_) of the designed product after oxidation upsurges from pH 3.0 to 5.0. At pH 5.0, the peak current is exploited (Figure 3 inset) and then, at advanced pH values, the current drops suddenly. This supports the expectable actions of protons in a proton-dependent manner and irreversibility. As LV has a pKa value of 6.02 for carboxylic groups and 8.74 for piperazinyl groups [29], the selected pH value of 5.0 makes its detachment and communication with the NFS easier in the area close to the first detachment of LV.

#### 3.3.2. Influence of Scan Rate

**Figure 4:**
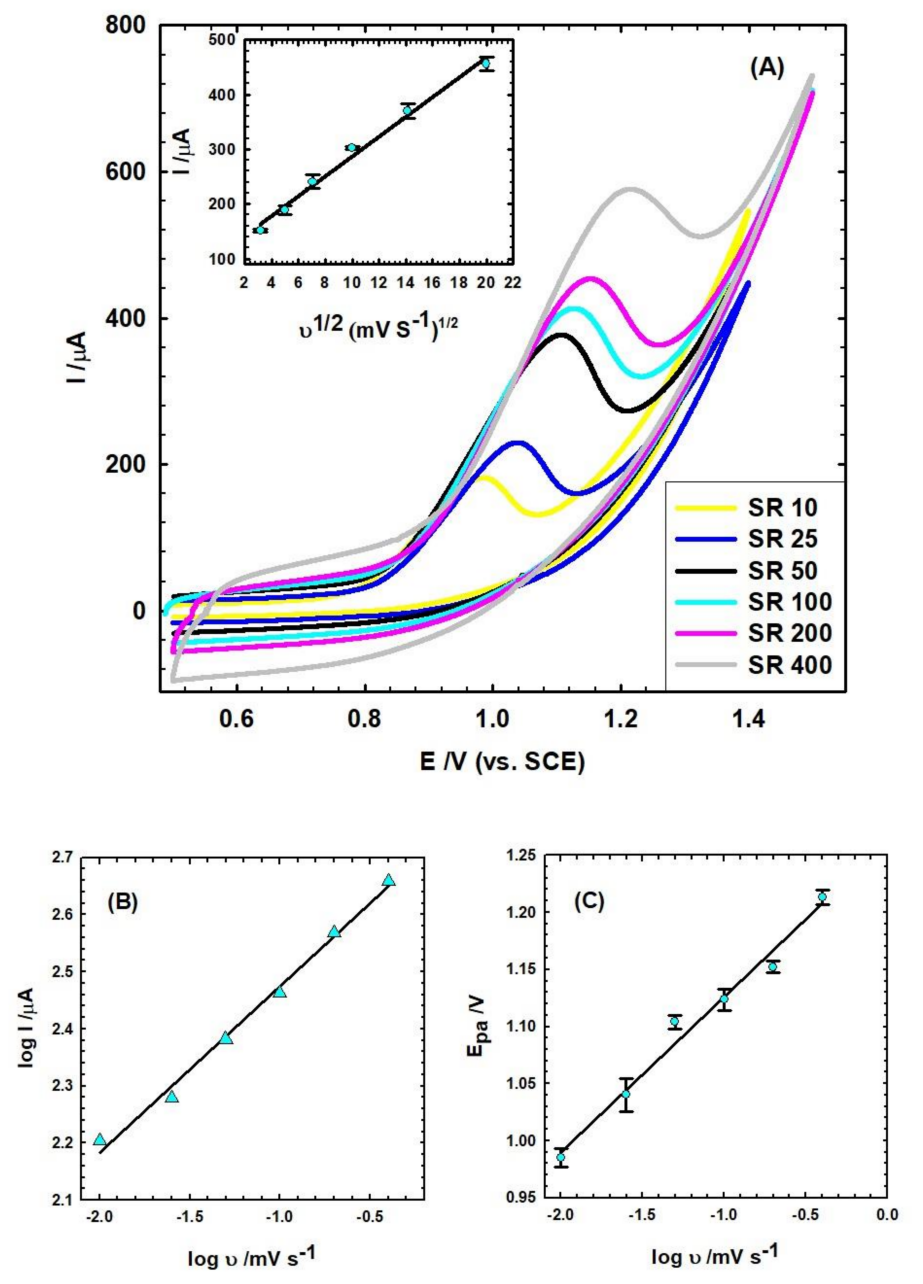

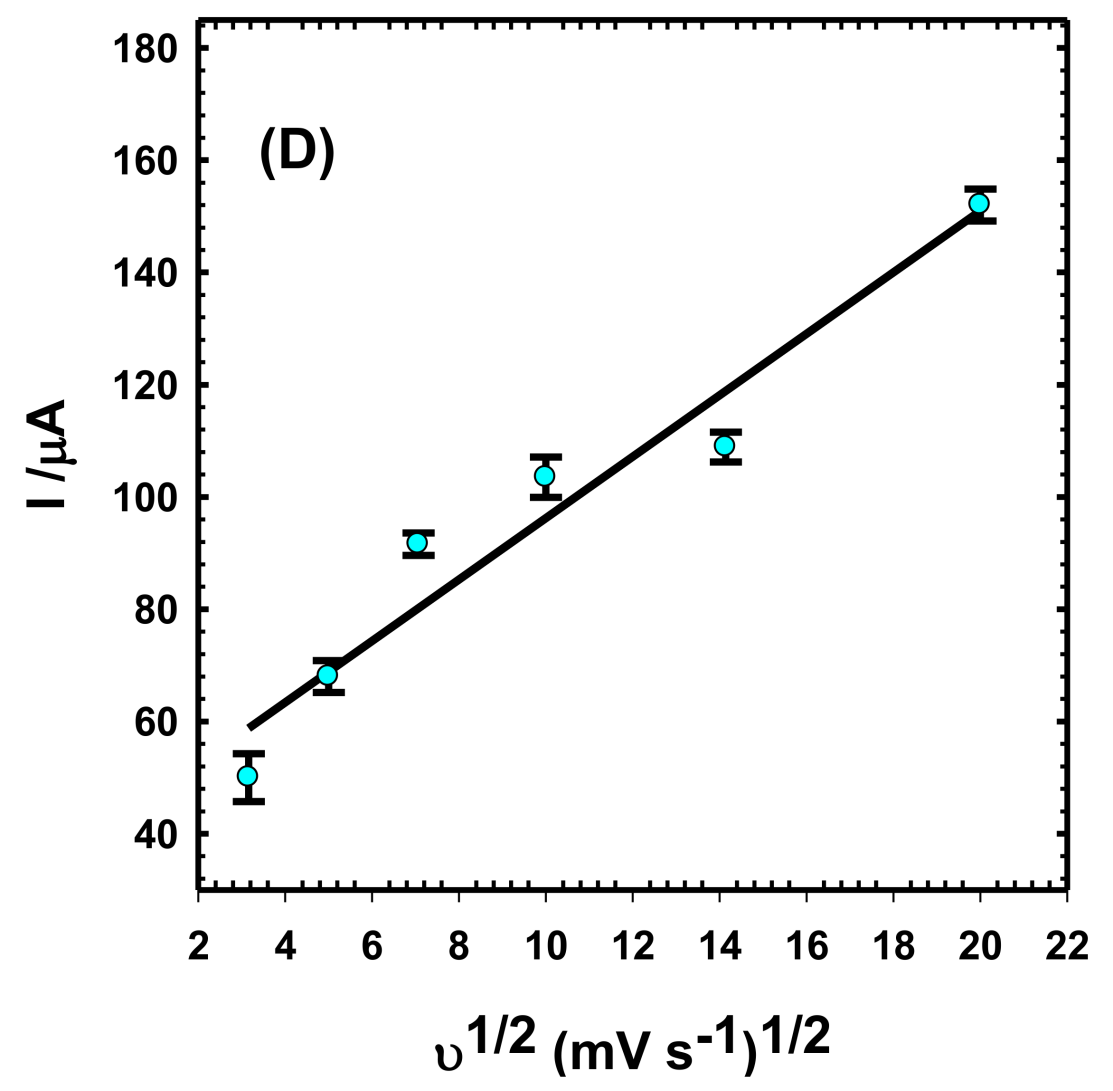
(**A**) CVs of 1.0 mM LV in BR (pH 5.0) by means of various SRs (10.0–400 mV s^−1^). Inset: The I_pa_ against the square root of SR at NFS/CPE. (**B**) The logarithm of the I_pa_ and the logarithm of the SR at NFS/CPE. (**C**) The E_pa_ against the logarithm of the SR at NFS/CPE. (**D**) The I_pa_ against the square root of SR at CPE.

An examination of the SR alteration on the I_pa_ of 1.0 mM LV was accomplished by CV (Figure 4A). The relationship between I_pa_ and the square root of the SR (ν = 10 to 400 mV/s) provides a linear relation, as identified in the insertion of Figure 4A, indicating a diffusion-controlled mechanism [18,27]. The equation is Ip(μA) = 18.14 ν^1/2^ + 105.6 (R^2^ = 0.99) for the NFS/CPE and Ip(μA) = 5.47 ν^1/2^ + 41.5 (R^2^ = 0.94) for the CPE (Figure 4D). The relation between log I and log ν for the NFS/CPE is linear (log Ip(μA) = 0.29 log ν + 2.77 (R^2^ = 0.99)), with a slope of 0.29, (Figure 4B), which confirms that the oxidation process of LV is a diffusion-controlled mechanism. Furthermore, the E_p_ of LV is moved to the positive path with a growing SR (Figure 4C), signifying irreversibility with the following equation: E_pa_(V) = 0.14 log ν + 1.26 (R^2^ = 0.97).

According to the Randles–Sevcik equation [17,18], I_p_ = 2.69 × 10^5^ n^3/2^ AD^1/2^ ν ^1/2^C, where Ip is the peak current (A), n is the quantity of electrons transported, ν is the SR (V s^−1^), D is the diffusion coefficient, A is the area (cm^2^), and C is the concentration. For 1.0 mM K_4_Fe(CN)_6_ in 0.10 M KCl, n is 1 and D is 7.6 × 10^−6^ cm s^−1^. The active surface area of CPE is 0.1 cm^2^ and that of the NFS/CPE is 0.34 cm^2^. It is clear that NFS as a modifier upsurges the active surface area 3.4 times more than in the case of a bare CPE.

### 3.4. Influence of Accumulation Time

**Figure 5:**
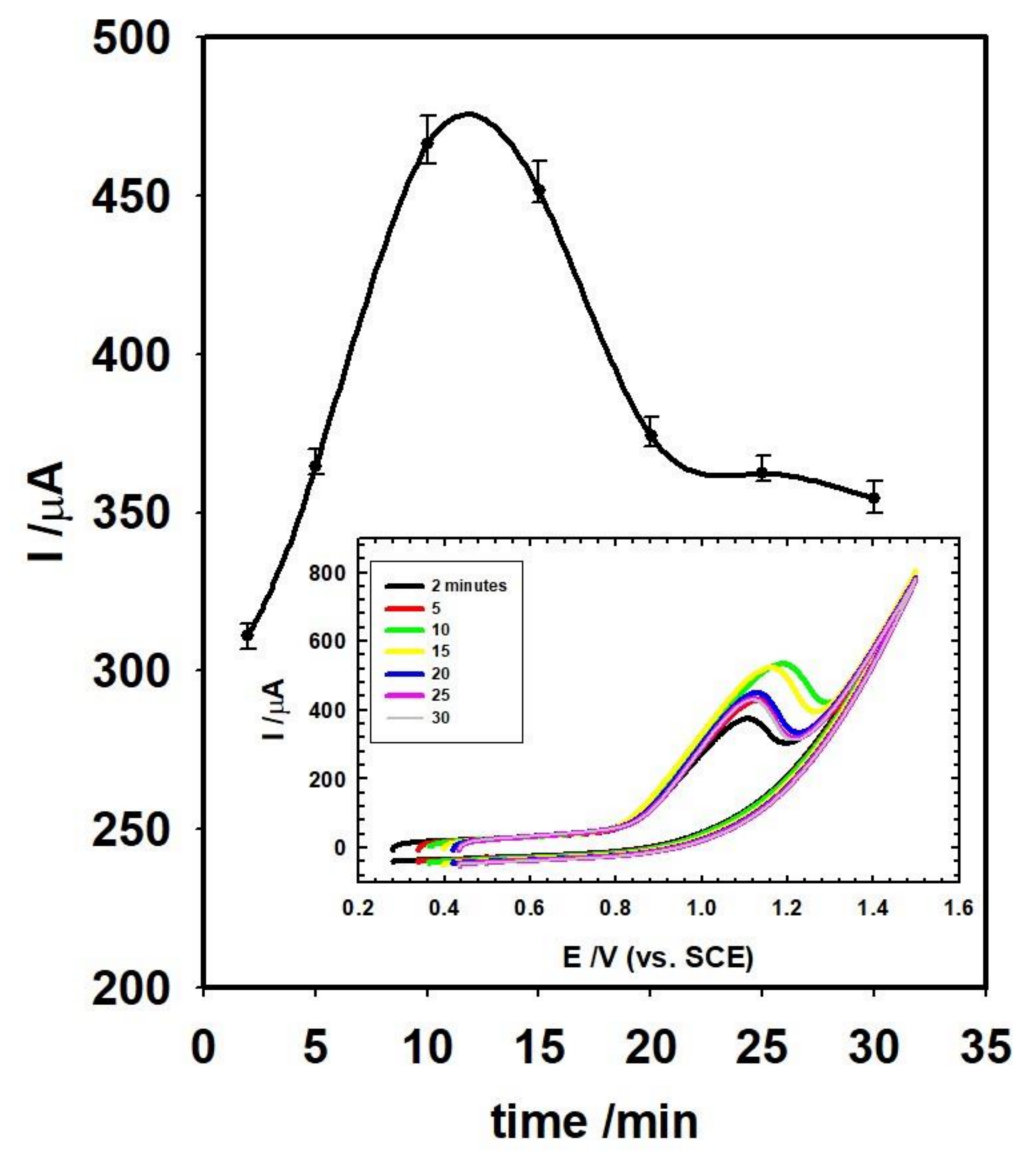
Outcome of the accumulation of time on I_pa_ for 1.0 mM of LV in BR (pH 5.0) at NFS/CPE. Inset: the corresponding data as CVs.

The CVs for the NFS/CPE electrode were measured for 1.0 mM of LV in BR (pH 5.0) at a scan rate of 50 mV/s as a function of time (30 min). It was found that time significantly affects the peak current value, where it first increases until 12 min and then decreases, and after 20 min of immersion, it reaches a plateau. The accumulation of time has a significant impact on the electrochemical response of the NFS/CPE when detecting 1.0 mM of LV in BR (pH = 5.0), as it influences the amount of LV adsorption on the electrode surface [5,18,30]. Figure 5 shows that the peak current clearly upsurges until nearly 10 to 15 min, owing to the equilibrium of LV on the NFS/CPE surface. To achieve a good sensitivity, speed, and equilibrium, an accumulation time of 12 min was employed. After 12 min, the signal intensity decreases sharply owing to the adsorption of the oxidation product and complexation [31,32] on the surface, and no more active sites are available on the electrode’s surface, that is, the surface is blocked.

### 3.5. Calibration Curve and Detection Limit

**Figure 6:**
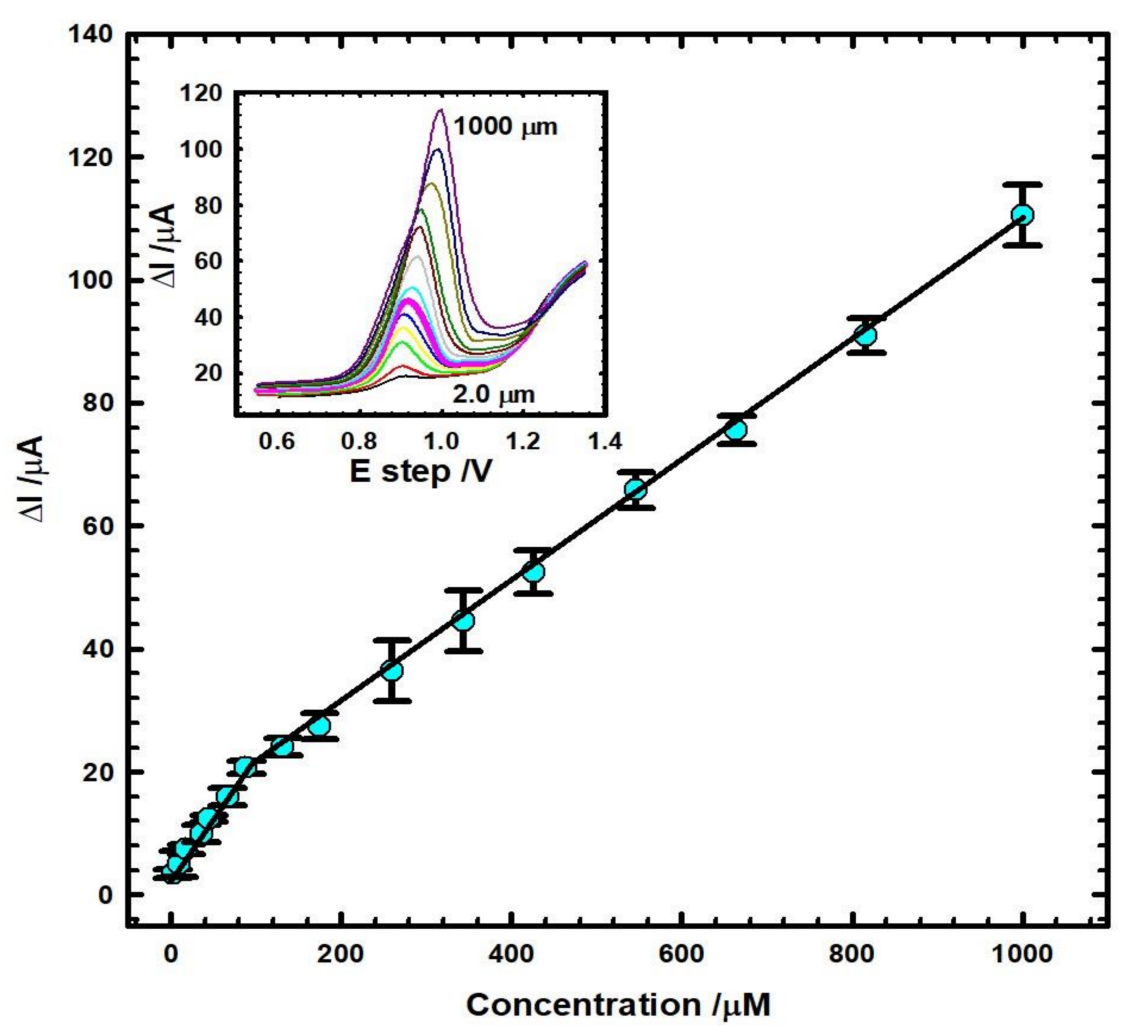
Calibration curve of LV using NFS/CPE.

The Figure 6 inset demonstrates the DPV for altering the LV concentration (2.0 to 1000 µM) in BR buffer (pH 5.0) after stirring for 12 min at a SR of 10 mV/s by means of the NFS/CPE. The response of levofloxacin was linear in two concentration series (2.0–88 µM and 131.6–1000 µM) using the improved electrode. Under optimal settings, the relation was linear, as presented in Figure 6: Ip(µA) = 0.20 C + 3.19, R^2^ = 0.98 and Ip(µA) = 0.099 C + 10.52, R^2^ = 0.99. The limits of detection (LOD = 3 σ/S) and quantification (LOQ = 10 σ/S) were 0.09 µM and 0.30 µM, correspondingly, where S is the calibration curve slope (0.099) and σ is the standard deviation of the blank (2.97 × 10^−3^) [25,33,34]. The repeatability of the anticipated electrode was inspected by reiterating the tests five times under the matching circumstances, generating a relative standard deviation (RSD) of 1.31%.

Inset: DPV of consecutive adding of LV in BR buffer (pH 5.0) by means of NFS/CPE at a SR 10 mV/s.

As revealed in Table 1, the high responsiveness of the NFS/CPE in terms of LV detection was confirmed and compared to others.

### 3.6. Commercial Samples Analysis

The applicability of the sensor was verified by identifying LV in real samples by DPV in a standard addition manner. The outcomes are presented in Table 2 and show that the electrode can be used with acceptable recoveries. Each measurement of peak oxidation currents was performed an average of five times to explore LV in the real samples.

### 3.7. Interference Study, Selectivity, Reproducibility, and Stability of NFS/CPE

**Figure 7:**
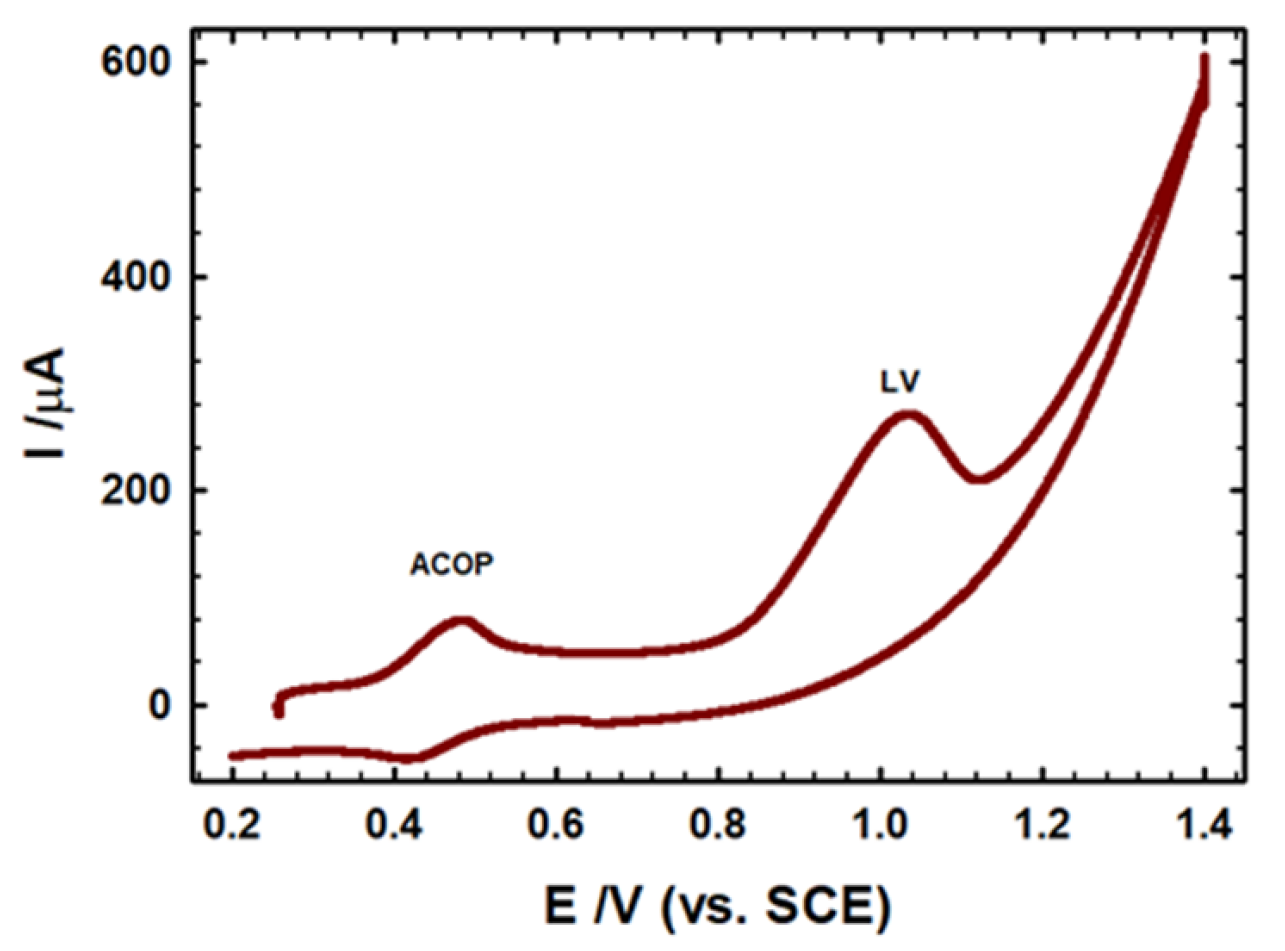
Instantaneous determination of 6 × 10^−4^ M LV and ACOP.

To see the impact of altered interfering compounds on the discovery of LV by the NFS/CPE, a constant concentration of LV (1.0 mM) was spiked with equivalent (1.0 mM) and doubled (2.0 mM) concentrations of several compounds such as glucose, sucrose, starch, and urea. The normal reference concentration range for urea in the blood is 1.8 to 7.1 mM [41]. Herein, I used a spiked concentration of 1.0 and 2.0 mM of urea as an interfering material, which is lower than or in the range of values with no noticeable interference with LV. 0.6 mM, and its doubled concentration was used as an interfering material, with the reference therapeutic dose of paracetamol being 0.05 to 0.1 mM [22]. The experiments at low concentrations were carried out in the mentioned range with no peaks or any interference appearing. However, on using higher concentration levels (0.6 mM) of paracetamol, a sharp and separated peak was obtained, as shown in Figure 7, without any interference with LV. This confirms the sensitivity of the proposed electrode towards levofloxacin in the given potential range.

By means of identical experimental circumstances, CV is carried out and the outcomes are presented in Table 3. It is seen that the relative sensor response decreases in all cases when doubled concentrations are used. This is because these interfering materials share functional groups with the LV drug structure, such as hydroxyl groups in glucose, sucrose, starch, and carbonyl groups, as well as nitrogen atoms in urea.

To verify the reproducibility of the NFS/CPE, four different electrodes were analyzed in 1.0 mM LV and an RSD value of 2.3% was obtained, ensuring the precision of the electrode. The stability of the planned system was assessed by keeping the NFS/CPE at laboratory temperature for 12 days. It was found that 94% of the initial peak current was reserved.

### 3.8. Instantaneous Determination of LV and Paracetamol

Paracetamol (ACOP) is habitually taken with LV in various anticipated protocols [17,22,23]. Thus, the examination of the selectivity of the NFS/CPE sensor for LV is mandatory. An instantaneous determination of 6 × 10^−4^ M LV and ACOP in BR buffer (pH 5.0) with the NFS/CPE was accomplished. Figure 7 shows good distance between ACOP (at 480 mV) and LV (at 1027 mV) with an ΔE of 547 mV, designating the electrocatalytic activity of LV and ACOP in the simultaneous presence of both. Thus, this sensor is selective for both ACOP and LV due to the fact that both drugs are always used together for flu or COVID-19. Recently, ACOP was used to reduce fevers and control inflammation in SARS-COVID-19 patients. Thus, its detection is extremely topical compared with other drugs. Thus, it is important to measure both LV and ACOP at the same time to ensure sensor selectivity.

## 4. Conclusions

An innovative CPE synthesized with nano-sized fumed silica provided exceptional performance for the electrochemical determination of LV. The peak current value (I_p_) of the NFS/CPE sensor was found to be 2.7 times greater compared to that of the bare electrode, which means it had an excellent response. Electrochemical impedance spectroscopy (EIS) was performed at a peak potential (E_p_) of +1066 mV and gave a very low resistance value of 10 kΩ for the newly created NFS/CPE sensor compared to 2461 kΩ for the bare electrode. This indicates the NFS/CPE’s high conductivity and confirms its high response to currents. Both techniques indicate a high conductivity with regard to the newly synthesized sensor. Thus, nano-sized fumed silica significantly improved the responsiveness to LV. The detection limit was 0.09 µM. Also, the interference of excipients with the drugs did not affect the detection of LV. The outcomes revealed that the NFS/CPE is simple, cheap, and easy to use and sensitive enough for LV determination in Quinostarmax tablets and human plasma, with a respectable precision and a small detection limit.

## Figures and Tables

**Table 1:** Analytical parameters comparison of several electrodes for LV determination with this work.

Modified Electrode	Linear Range (µM)	LOD (µM)	Reference
PoAP/MWCNTs/GCE	3.0–200	1.0	[35]
Au/PDDA/rGO/GCE	10–800	3.9	[36]
Azure-B/PGE	2–125	1.2	[37]
DsDNA/GCE	0.5–5	0.1	[38]
MWCNT-SnO_2_/GC	1–9.9	0.2	[39]
MIP/G-AuNPs	1–100	0.53	[40]
NFS/CPE	2–1000	0.09	This work

PoAP = poly(o-aminophenol), MWCNTs = multiwalled carbon nanotubes, Au = gold nanoparticles, PDDA = poly(diallyldimethylammonium chloride), rGO = reduced graphene oxide, PGE = pencil graphite electrode, GCE = glassy carbon electrode, GE = graphite electrode, CPE = carbon paste electrode, MIP = molecularly imprinted polypyrrole, G-AuNPs = graphene–gold nanoparticles.

**Table 2:** Recoveries of LV in human plasma and pharmaceutical samples at pH 5.0 (*n* = 5).

LV Added (µM)	Found (µM)	Recovery (%)	RSD (%)
Human plasma			
5	4.80	96.0	2.6
10	9.52	95.2	1.9
15	14.62	97.5	2.2
Pharmaceutical samples			
8.88	9.81	110.47	2.3
17.75	19.2	108.17	1.9
35.43	34.45	97.23	1.5
44.25	44.38	100.29	2.1
88.11	88.07	99.95	1.6

RSD (%) was calculated for the average of five trials.

**Table 3:** The influence of interfering materials by NFS/CPE.

Interfering Material	Relative Sensor Response (%) *	
	1:1	1:2
Glucose	97.1	96.3
Sucrose	97.3	96.1
Starch	95.8	95.2
Urea	97.0	96.5
Paracetamol	99.8	99.5

* Average of five measurements.

## Data Availability

The raw/processed data required to reproduce these findings cannot be shared at this time as the data also forms part of an ongoing study.
